# CALHM1-Mediated ATP Release and Ciliary Beat Frequency Modulation in Nasal Epithelial Cells

**DOI:** 10.1038/s41598-017-07221-9

**Published:** 2017-07-27

**Authors:** Alan D. Workman, Ryan M. Carey, Bei Chen, Cecil J. Saunders, Philippe Marambaud, Claire H. Mitchell, Michael G. Tordoff, Robert J. Lee, Noam A. Cohen

**Affiliations:** 1 Perelman School of Medicine at the University of Pennsylvania Philadelphia, Philadelphia, PA USA; 2 Department of Otorhinolaryngology: Head and Neck Surgery, University of Pennsylvania, Philadelphia, PA USA; 3 Feinstein Institute for Medical Research, Manhasset, NY USA; 4 Department of Anatomy & Cell Biology, University of Pennsylvania School of Dental Medicine, Philadelphia, PA USA; 5 Monell Chemical Senses Center Philadelphia, Philadelphia, PA USA; 6 Division of Otolaryngology: Head and Neck Surgery, Philadelphia Veterans Administration Medical Center, Philadelphia, PA USA

## Abstract

Mechanical stimulation of airway epithelial cells causes apical release of ATP, which increases ciliary beat frequency (CBF) and speeds up mucociliary clearance. The mechanisms responsible for this ATP release are poorly understood. CALHM1, a transmembrane protein with shared structural features to connexins and pannexins, has been implicated in ATP release from taste buds, but it has not been evaluated for a functional role in the airway. In the present study, *Calhm1* knockout, *Panx1* knockout, and wild-type mouse nasal septal epithelial cells were grown at an air-liquid interface (ALI) and subjected to light mechanical stimulation from an air puff. Apical ATP release was attenuated in *Calhm1* knockout cultures following mechanical stimulation at a pressure of 55 mmHg for 50 milliseconds (p < 0.05). Addition of carbenoxolone, a PANX1 channel blocker, completely abolished ATP release in *Calhm1* knockout cultures but not in wild type or *Panx1* knockout cultures. An increase in CBF was observed in wild-type ALIs following mechanical stimulation, and this increase was significantly lower (p < 0.01) in *Calhm1* knockout cultures. These results demonstrate that CALHM1 plays a newly defined role, complementary to PANX1, in ATP release and downstream CBF modulation following a mechanical stimulus in airway epithelial cells.

## Introduction

Effective mucociliary clearance, achieved through the coordination and modulation of ciliary beating, plays a critical role in respiratory defense. The cilia propel an airway surface liquid (ASL) that traps aerosolized debris and pathogens to the oropharynx, where it can be eliminated by either swallowing or expectoration^1^. Individuals who have immotile or dysfunctional cilia, such as those with primary ciliary dyskinesia or cystic fibrosis, are predisposed to frequent respiratory infections^2, 3^. When cilia are working properly, even small increases in ciliary beat frequency (CBF) can dramatically speed up clearance of the ASL^4^.

Several mechanisms exist in ciliated cells of the respiratory system to tightly control CBF and maintain efficiency. Many of these regulatory pathways result in increased extracellular ATP in the airway lumen^5^, which in turn increases the intracellular free Ca^2+^ concentration^6–8^, which subsequently results in an increase in CBF. Specifically, ATP binds two types of purinergic receptors, P2X and P2Y, on the apical surface^7, 9, 10^, with each receptor serving a unique purpose in modifying intracellular Ca^2+^. P2X receptors cause Ca^2+^ influx via ATP-gated Ca^2+^ permeable channels, and P2Y receptors cause Ca^2+^ release from intracellular stores via heterotrimeric G-protein activation of phospholipase C and the generation of inositol 1,4,5-trisphosphate (IP_3_)^11–14^. Following ATP stimulation of either receptor, the resulting free Ca^2+^ in the cytoplasm enhances CBF^15, 16^. Apyrase, a compound that degrades extracellular ATP, abolishes CBF increases following mechanical stimulation^17^.

The release of endogenous ATP from the cytoplasm to the airway surface involves pathways that are activated by mechanosensors on the ciliated cells^18^. In addition, forces generated by breathing, sneezing, or coughing result in ATP release from airway epithelial cells^19–21^. Other mechanical changes, such as liquid shear stress or hypotonicity, also reliably increase extracellular ATP concentration^22–24^. The mechanisms that mediate this ATP release have not yet been fully elucidated, but may include exocytotic release of vesicular ATP^25^ and ion channels, including connexin hemichannels and pannexins^26–28^.

PANX1 (pannexin 1) is a relatively non-selective ion channel^26^ that has been shown to mediate hypotonic stress and cellular deformation-induced ATP release in airway epithelium^18^. Pannexin channel blockers or siRNA knockdown of *Panx1* inhibit ATP release by ~60% in response to hypotonic cell swelling in ciliated epithelial cell cultures^29^. Membrane stretch triggers Ca^2+^-independent ATP release through PANX1 channels^26, 30^, which is believed to play a role in local signaling to coordinate CBF increases^31, 32^. Carbenoxolone, a broad spectrum PANX1 channel blocker, inhibits ATP release by 54% from the nasal mucosa in response to physical deformation, while not completely abolishing the response^33^. The ATP release that is observed in airway epithelial cells even when PANX1 is blocked^18^ suggests that redundant pathways might contribute to ATP release following mechanical stimulation.

Calcium Homeostasis Modulator 1 (CALHM1) is a non-selective, weakly voltage gated Ca^2+^ permeable ion channel that shares structural features with connexins and pannexins^34^. The pore of CALHM1 is ~14 Å wide, similar to that of connexins^34^. It is activated in part by membrane depolarization and by decreases in extracellular Ca^2+^ concentration^35^. Originally discovered in an Alzheimer’s disease paradigm^36, 37^, CALHM1 plays a role in cortical neuron excitability^35^ as well as in peripheral taste perception^38^. Mice that lack the *Calhm1* gene show no avoidance of bitter compounds and no preference for sweet and umami compounds because CALHM1 mediates the release of ATP from type II taste receptor cells in response to these compounds^38^. CALHM1-mediated ATP release is inhibited by ruthenium red, but not by heptanol or carbenoxolone, which block connexins and PANX1, respectively^39^.

*Calhm1* is expressed in taste buds and in the brain, as well as in a variety of other human tissues, including the trachea^36^. We considered that CALHM1 might contribute to ATP release in the airway as other taste mediated signaling proteins have been identified in respiratory epithelial cells^40, 41^. In the present study, we confirmed *Calhm1* gene expression in airway epithelium and investigated the role of CALHM1 in ATP release in airway epithelial cells in response to mechanical stimulation that simulates a sneeze.

## Results

### ATP Release Following a 55-mmHg Air Puff

Prior work established that a 55-mmHg air puff for 50 milliseconds simulates the pressures and timing of a sneeze, and that this is an effective *in vitro* model of physiologic dynamic regulation of ciliary beat frequency^17^. In wild type Air-Liquid Interface (ALI) cultures, CBF is reliably increased via this mechanical stimulation, and the magnitude of the CBF increase correlates with the pressure delivered to the apical surface^17, 42^. Mechanical stimulation of CBF involves ATP release from the cells, purinergic stimulation, and subsequent increase in intracellular Ca^2+^ concentration and Protein Kinase A (PKA) activation^17^.

**Figure 1:**
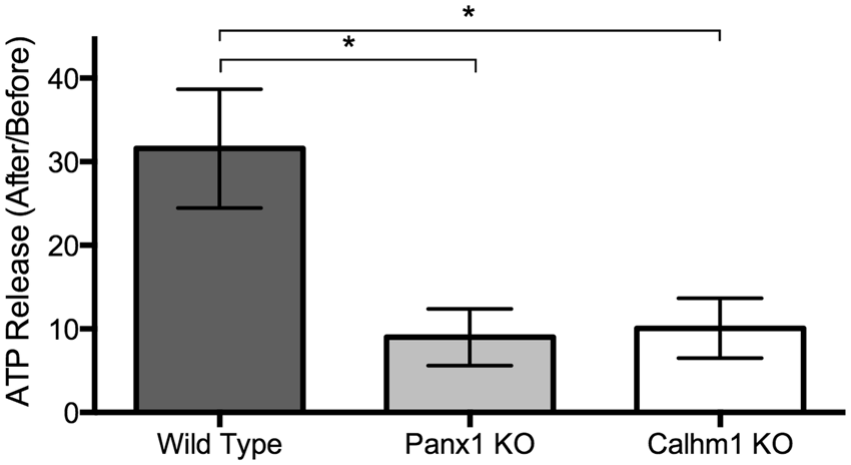
Apical ATP release 15 seconds after a 55-mmHg air puff in nasal septal epithelial cell cultures from *Calhm1* knockout, *Panx1* knockout, and wild-type mice. Bars are means ± SE of 5 cultures each. *p < 0.05, ANOVA with Dunnett’s multiple comparisons test.

Immediately following a 50-millisecond 55-mmHg air puff, culture airway surface liquid was collected, and ATP quantified. Apical ATP concentration increased in wild-type cultures 31.6-fold (baseline ATP 0.19 ± 0.02 μM), but only 10-fold in *Calhm1* knockout cultures (baseline ATP 0.31 ± 0.04 μM) and 7-fold in *Panx1* knockout cultures (baseline ATP 0.55 ± 0.08 μM) (n = 5 cultures per condition, p < 0.05, ANOVA with Dunnett’s multiple comparisons test; Fig. 1). This differential ATP release suggests that CALHM1, in addition to the previously studied ATP release channel PANX1, contributes to respiratory epithelial ATP release following mechanical stimulation.

**Figure 2:**
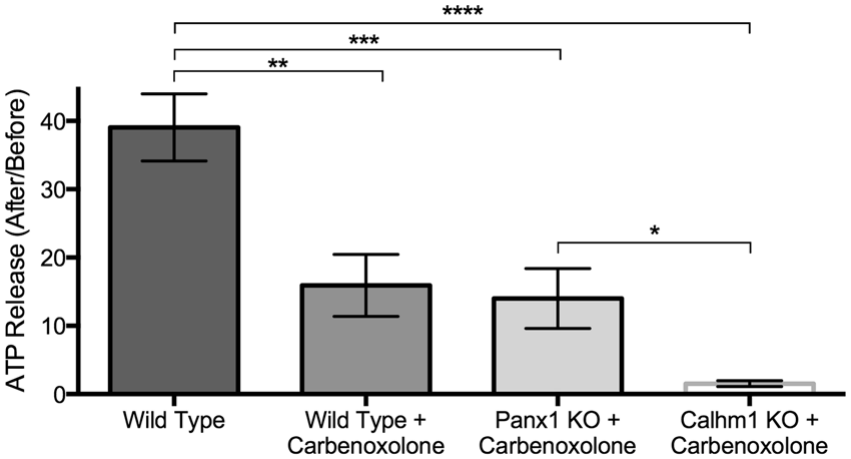
Apical ATP release 15 seconds after a 55-mmHg air puff in nasal septal epithelial cell cultures from wild-type (control) mice, and *Calhm1* knockout, *Panx1* knockout, and wild-type mice preincubated with 150 μM carbenoxolone. Bars are means ± SE of 5–6 cultures each. *p < 0.05, **p < 0.01, ***p < 0.001, ****p < 0.0001, ANOVA with Holm Sidak’s multiple comparisons test.

Carbenoxolone is a PANX1 channel blocker that has been shown to have no activity in blocking CALHM1 currents^43^, but is not specific for PANX1 as it blocks other channels, including gap junctions. We incubated wild-type, *Calhm1* knockout, and *Panx1* knockout ALI cultures with 150 μM carbenoxolone for 20 minutes prior to stimulation, a concentration and time period that has been previously used for successful PANX1 blockade^44^. Apical ATP concentration increased 39 fold in wild type cultures (baseline ATP 0.36 ± 0.09 μM), 16-fold in wild type cultures incubated with carbenoxolone (baseline ATP 0.29 ± 0.09 μM), 14-fold in *Panx1* knockout cultures incubated with carbenoxolone (baseline ATP 0.25 ± 0.06 μM), and 1.5-fold in *Calhm1* knockout cultures incubated with carbenoxolone (baseline ATP 0.36 ± 0.09 μM) (Fig. 2). These differences were significant (p < 0.0001, ANOVA with Holm-Sidak’s multiple comparison test). These results demonstrate that for ATP release, carbenoxolone does not have an apparent effect beyond blocking PANX1, and that *Calhm1* knockout cultures with blocked PANX1 (by pre-incubation with carbenoxolone) have completely abolished ATP release in response to mechanical stimulation.

### Calhm1 and Ciliary Beat Frequency Modulation

**Figure 3:**
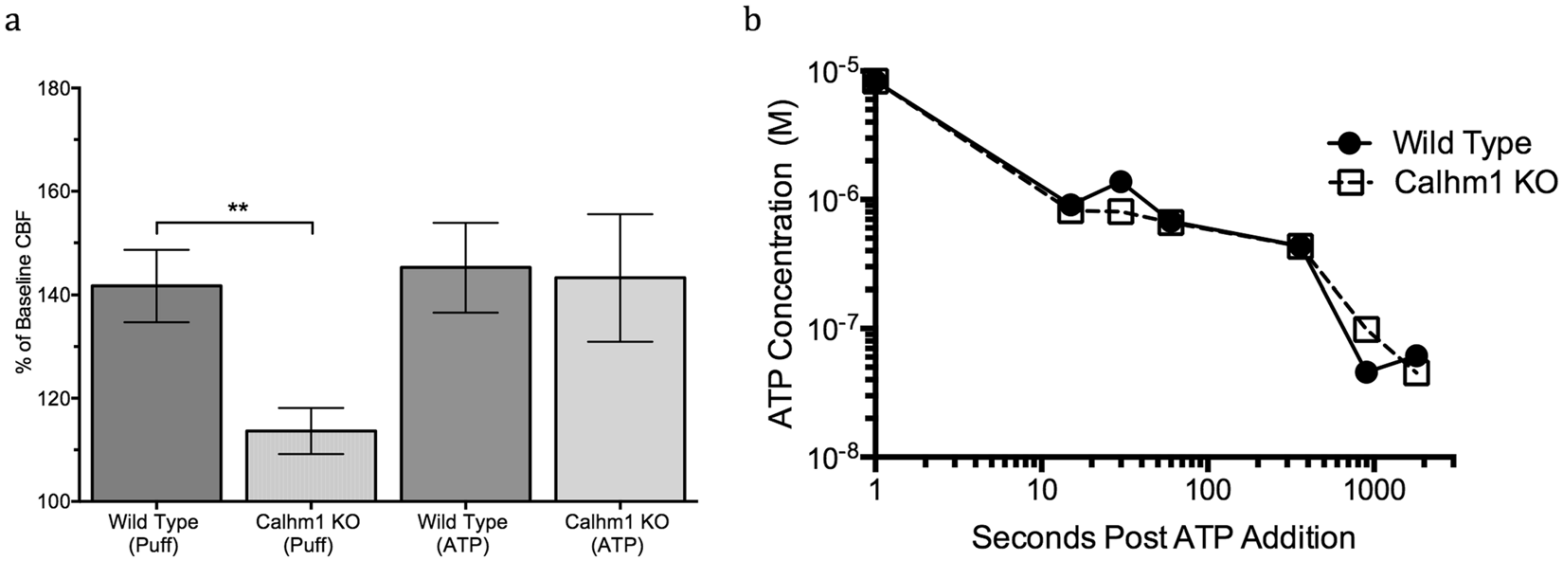
(**a**) CBF Increases 15 seconds after 1-μM ATP addition or 55-mmHg air puff in nasal septal epithelial cell cultures from *Calhm1* knockout and wild-type mice. Bars are means ± SE of 3–6 cultures each. (**b**) ATP degradation in *Calhm1* knockout wild-type mice. 5 × 10^−7^ M ATP was added to the apical surface t = 0, and apical ATP concentration was measured at six distinct time intervals following this addition. All Apical ATP concentrations are adjusted for a 3.6 μl ASL volume.

While the role of PANX1 as an airway ATP release channel has been well documented^45^, the presence and function of CALHM1 in the airway has not been established. Previous studies have demonstrated that PANX1 blockade attenuates ciliary beat frequency increases, which are driven by ATP release^46^. To further investigate the properties of the CALHM1 ATP release pathway, we asked whether the reduced ATP observed in the *Calhm1* knockout cultures was also accompanied by a reduced CBF. Prior experiments have demonstrated that maximal changes in CBF occur at 15 seconds following mechanical stimulation^17^. In response to a 50 millisecond 55-mmHg air puff, CBF of wild-type cultures (n = 12, baseline 12.4 ± 0.9 Hz) increased above baseline frequency at 15 seconds post-stimulation, while in the *Calhm1* knockout cultures (n = 12, baseline 8.7 ± 1.2 Hz), the CBF increase was significantly smaller (Fig. 3a) (p < 0.01, two-tailed t-test). The difference in baseline CBF frequency was also statistically significantly different between the two conditions (p < 0.05, two-tailed t test). While ATP release is an early event in the transduction of mechanical stimulation signals, gap junctions (which are blocked by carbenoxolone) are necessary downstream to increase CBF^47^. Supplemental Fig. 1 further supports this hypothesis, as all carbenoxolone pre-incubated cultures (wild type and *Calhm1* knockout) show no CBF changes when stimulated with a 55 mmHg air puff.

To determine whether the reduced ATP release contributed to reduced CBF in the *Calhm1* knockout cultures, 30 μl of 2-μM ATP dissolved in PBS was introduced to the apical surface of three wild-type cultures and three *Calhm1* knockout cultures. Each culture had 30 μl of PBS on the apical surface before the experiment began, resulting in a final corrected ATP concentration (adjusted for an assumed ASL volume of 3.6 μl) of 16 μM, consistent with a physiologic post-stimulation ATP concentration. ATP was added at t = 0 seconds, with the post-addition recording occurring at t = 15 seconds. CBF in wild-type cultures (baseline 13.4 ± 3.7 Hz) and *Calhm1* knockout cultures (baseline 13.8 ± 3.8 Hz) increased similarly at 15 seconds (Fig. 3a). Thus, ATP-dependent CBF responses were intact in the *Calhm1* knockout cultures, suggesting that reduced ATP release contributed to reduced CBF responses to mechanical stimulation in the *Calhm1* knockout cultures.

The differences observed in CBF changes between *Calhm1* knockout and wild-type mice following mechanical stimulation could also be accounted for by differential degradation of ATP between the two types of cultures, so we set out to demonstrate equivalence in ATP degradation rates. To test this, 0.5 μM ATP was added to the apical surface of the cell cultures, and apical samplings were aspirated at six distinct time intervals (3 per culture). At all time points (15 seconds, 30 seconds, 1 minute, 5 minutes, 15 minutes, 30 minutes), there was no difference in ATP degradation rate between the cultures (Fig. 3b).

### Depolarization and CBF Changes

**Figure 4:**
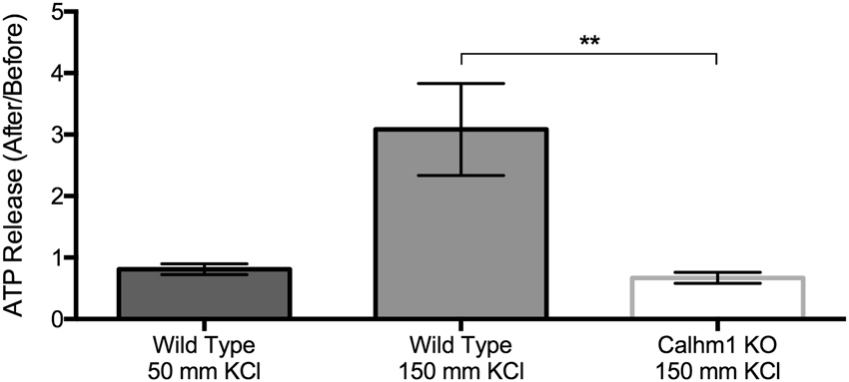
Apical ATP release 15 seconds after addition of 50 or 150 mM KCl in wild type and *Calhm1* knockout cultures. Bars are means ± SE of 3–5 cultures each. *p < 0.05, **p < 0.01, Kruskal-Wallis with Dunn’s multiple comparisons test.

CALHM1 is a weakly voltage-gated channel^35^, so we set out to test if KCl in a known depolarizing concentration^48^ would induce ATP release in ALI cultures. Addition of 50 mM KCl (control) produced no significant ATP release in wild-type cultures, while addition of 150 mM KCl increased ATP concentration on the apical surface 4-fold in wild type cultures, which was significantly greater than ATP changes seen in *Calhm1* knockout cultures (n = 3–5 cultures per type, p < 0.01, Kruskal-Wallis with Dunn’s multiple comparison test; Fig. 4). This increase in ATP release was a full order of magnitude smaller than that observed with mechanical stimulation in our previous experiments.

### Absence of Cellular Lysis following a 55 mmHg Air Puff

To test the hypothesis that cellular lysis following a 55 mmHg air puff was driving cellular ATP release, as opposed to ATP release through CALHM1, we performed a lactate dehydrogenase (LDH) assay following cellular stimulation (Supplemental Fig. 2). LDH levels were similarly low both in the control (PBS) and 55 mmHg air puff condition, indicating minimal cellular lysis. In an air puff of doubled strength (110 mmHg), significant release of LDH was observed above control and 55 mmHg conditions (n = 3 cultures per condition, p < 0.05, ANOVA with Dunnett’s multiple comparison test).

### qPCR for Calhm1 and Panx1 in Cells Grown at an Air-Liquid Interface

Using cDNA obtained from *Panx1* knockout, Calhm1 knockout, and wild-type mouse cultures, real-time qPCR was performed to confirm *Calhm1* and *Panx1* gene expression in the ALI cultures. Additionally, we confirmed the relative expression levels of *Calhm1* in respiratory epithelium of wild type and *Panx1* knockout mice, and the relative expression levels of *Panx1* in wild type and *Calhm1* knockout mice. 3–6 replicates using β-actin and 3–6 replicates using target primers *(Calhm1/Panx1)* were performed on each cDNA sample, normalizing *Calhm1/Panx1* expression to β-actin in order to determine relative expression in each condition. *Calhm1* expression levels were not significantly different in *Panx1* knockout and wild-type mice, and *Panx1* expression levels were not significantly different in *Calhm1* knockout and wild-type mice (Supplemental Fig. 3A and B). *Panx1* transcripts are present in wild type samples at approximately 100 times the level of *Calhm1* transcripts. Notably, no relative expression of target genes was observed in corresponding negative controls; there was no amplification of *Calhm1* transcripts in *Calhm1* knockout cultures or of *Panx1* transcripts in *Panx1* knockout cultures.

## Discussion

Mechanical stimulation of airway epithelial cells causes cellular ATP release and elevated intracellular Ca^2+^ concentration, which in turn increases CBF^16^, partially due to ATP-activated P2X and P2Y receptors that are modulatory^49–51^. Exogenously administered ATP increases CBF, and apyrase (which degrades extracellular ATP) has been previously shown to abolish the CBF increase following mechanical stimulation in an air puff paradigm^17^. However, the mechanisms that are involved in the initial ATP release from the cells are poorly defined. PANX1 is an ion channel that is known to mediate ATP release and CBF changes in the airway^46^, but PANX1 channel blockers or knockdown of *Panx1* only reduces this ATP release by approximately 50%^18, 33^, suggesting that additional channels are responsible for this persistent ATP release. CALHM1, a pore-forming channel that shares structural features with connexins and pannexins, has recently been shown to play an essential role in ATP release in taste receptor cells^38^. Of additional interest, several studies over the past 5 years have identified taste cell signaling molecules in ciliated cells of the respiratory epithelium^41, 52, 53^. Thus, we have investigated whether CALHM1 also has a role as the “missing channel” in modulation of airway ATP release. Quantitative PCR demonstrated that the *Calhm1* gene was expressed in respiratory cells grown at an ALI, which were used in all of the experiments.

After mechanical stimulation by a 55-mmHg pressure air puff, apical ATP release and subsequent CBF increases were significantly lower in *Calhm1* knockout cultures than in wild-type cultures. Exogenously applied ATP increased CBF to an equivalent extent in cultures derived from both *Calhm1* knockout and wild-type mice. Carbenoxolone, a PANX1 channel blocker that has no effect on the CALHM1 channel, completely abolishes ATP release following mechanical stimulation when applied to *Calhm1* knockout cultures, while leaving residual (presumably CALHM1-mediated) ATP release intact when applied in *Panx1* knockout cultures.

Thus, our findings are consistent with the hypothesis that CALHM1 plays a complementary and equally important role to Panx1 in ATP release following cellular stress or deformation of respiratory epithelial cells. CALHM1 also appears to be a critical channel in CBF modulation as a result of this ATP release. Wild type and *Calhm1* knockout cultures did not exhibit any difference in their metabolism of ATP, strengthening the evidence that channel release is driving the alteration in net apical ATP concentration. Of additional importance is that CALHM1 does not form gap junctions^54^, so we suspect that the CBF decrement observed in our knockout experiment is solely due to ATP release, and not due to propagation of the downstream signal.

It is known that CALHM1 is regulated by Ca^2+^ and membrane voltage to gate the channel, but complete understanding of the mechanism(s) has not been determined^35^. CALHM1 has also been localized to the endoplasmic reticulum^55^, where it could influence the intracellular Ca^2+^ homeostasis and signaling in the cell without being the specific pathway for apical ATP release in response to mechanical stimulation. CALHM1 has also not yet been demonstrated to be selectively present at the apical membrane of airway cells, so more work is needed to definitively demonstrate its unambiguous role in mechanically transduced ATP release. Further complicating the model is that pannexins exhibit properties of ATP-induced ATP release^45^, so a certain degree of initial ATP release, possibly through CALHM1, may be necessary for full recruitment of this channel. These pathways are not yet fully elucidated, but defining the role of CALHM1 in initiating ATP release is a critical first step.

CBF measurements and baseline ATP secretion in culture and in tissue explants are physiologic parameters with high levels of variability. Variations in culture age, degree of culture ciliation, and day-to-day changes in culture health can cause notable differences in baseline values for these physiologic parameters. To minimize any variability due to these factors, we ensured that control cultures matched their experimental counterparts in age and experimental timing. Despite this, baseline differences in CBF persist, even within a single culture. In one of our experiments, *Calhm1* knockout cultures had a statistically significantly lower baseline CBF than matched wild type cultures. This was likely reflective of day-to-day baseline variability and, based on our previous work with the mouse ALI, should have minimal effects on relative (%) change. However, it is also important to consider that the differences observed in baseline CBF could be due to effects of the CALHM1 channel. ATP release is also critical for mucin hydration, which can have effects on CBF^56^. While this would not affect our experimental paradigm, in which ASL was removed prior to experimentation, it is nonetheless an important consideration. However, the *Calhm1* knockout mice showed no evidence of phenotypic mucus plugging, nor was there any evidence of inadequate ASL hydration in ALI culture.

The discovery of a functional role for CALHM1 outside of the brain and taste buds suggests that the channel may have yet undiscovered functions in additional tissue types. The presence of CALHM1 in the airway, and its proposed role in mechanical signal transduction, has important implications for understanding regulation of mucociliary clearance and its dysregulation in diseases such as chronic rhinosinusitis. Human upper airway tissue from patients with chronic rhinosinusitis demonstrates a blunted stimulation of CBF when presented with a mechanical stimulus^17^, and this may play a critical role in the pathogenesis of the disease. Additionally, tissue from chronic rhinosinusitis patients also shows a blunted response following addition of exogenous ATP^57^. Reduced morbidity can be achieved in chronic rhinosinusitis patients by improving mucociliary movement, and subsequent clearance of pathogens, debris, and stagnant mucus. While exact functions are not fully known, it is possible that CALHM1 could be an important therapeutic target for this type of respiratory pathology.

## Methods

All protocols were reviewed and approved by the Research and Development Committee at the Philadelphia Veterans Affairs Medical Center and were carried out in accordance with both the University of Pennsylvania and The Philadelphia VA Medical Center guidelines for vertebrate animal studies from the Institutional Animal Care and Use Committee of the Philadelphia Veterans Affairs Medical Center.

### Reagents

The ATP, Carbenoxolone (CBX), and KCl used in these experiments were all purchased from Sigma-Aldrich (St. Louis, MO, USA). All Dulbecco’s PBS was supplemented with Ca^2+^ and Mg^2+^ using 100 mg/L of anhydrous CaCl_2_ and MgCl_2_-6H_2_O, respectively. For potassium chloride (KCl) addition experiments, all KCl was dissolved in PBS without NaCl and then osmolarity of the solution was adjusted to 290 mOsm with NaCl addition.

### Mice

Nasal specimens from *Calhm1* knockout mice^35, 36^, *Panx1* knockout mice^58^, and wild type littermates were obtained from breeding colonies maintained at the Monell Chemical Senses Center (Philadelphia, PA). *Panx1* knockout mice also had a passenger mutation in caspase 4/11. Both lines were maintained by backcrossing to C57BL/6J mice. Heterozygous mice were mated brother-to-sister to produce homozygous wild type and knockout mice for the experiments reported here.

### Air-Liquid Interface (ALI) Cultures

We previously described the culture of mouse nasal septal epithelium at an ALI^59^. Briefly, nasal septal epithelial cells were harvested from sinonasal complex specimens and were digested with collagenase and pronase. Cells were grown on Costar 6.5-mm transwell permeable filter supports (Corning Inc. Life Sciences, Lowell, MA, USA). For the first 7 days, the cells were fully submerged in culture medium, and subsequently fed from only the basal chamber. Cilia appeared within two weeks, and cultures were used within 3–5 weeks.

Unless otherwise noted, transwell inserts (#3470, Corning) were removed from media before experiments and placed in a transwell containing 600 μl of Hank’s Balanced Salt Solution (HBSS) with vitamins. 30 μl of PBS was added to the apical surface of the transwell insert unless a different volume was specified, and the cultures were allowed to adjust for 15 minutes to 26 °C to ensure a steady baseline ciliary beat frequency^60, 61^.

### Delivery of Air Puff

Air puff delivery to the ALI was performed as described by Zhao^17^, with one notable modification. Compressed air (Airgas Corp., Radnor, PA, USA) was released at the apical surface *via* a 22-gauge plastic catheter (BD, Franklin Lakes, NJ, USA) for 50 milliseconds, regulated by a Pico-Spritzer II 2-valve microinjector ejector (General Valve Corp., Fairfield, NJ, USA). In contrast with the experimental methods used by Zhao *et al*., the catheter was placed directly in the center of the transwell to deliver a more uniform stimulation across the transwell. The catheter was attached to a micromanipulator, which allowed for placement 6 mm above the apical surface, directly in the center of the transwell (3.25 mm away from each transwell side).

A manometer (Traceable Manometer/Pressure/Vacuum Gauge, 3460; Control Co., Friendswood, TX, USA) was used to calibrate the pressure delivered to the culture. The transwell insert was sealed with a stopper that had two 22-gauge holes, with pressure being applied through one hole while the other hole was connected to the manometer.

### CBF Imaging and Analysis

Ciliated areas in all cultures tested were observed using a 20X objective on an inverted microscope (Leica Microsystems, Bannockburn, IL, USA). A model A602f-2 Basler area scan high-speed monochromatic digital video camera (Basler AG, Ahrensburg, Germany) captured images at 100 frames/s with a resolution of 650 × 480 pixels. Digital image signals from the camera were sampled by an acquisition board (National Instruments, Austin, TX, USA) on a Dell XPS 710 workstation (Dell, Inc., Round Rock, TX, USA) running the Windows XP Professional operating system (Microsoft, Redmond, WA, USA). Video images were analyzed with the Sisson-Ammons Video Analysis (SAVA) software (National Instruments) that is specialized to quantify CBF^62^ by performing a whole-field analysis of the ciliated apical surface of the cultures, reporting a CBF that is the arithmetic mean of all of the cilia in the video field. Consistent with prior imaging analyses^17^, any videos in which less than 5% of the imaged field was ciliated were excluded. In reporting CBF values, changes were normalized per convention to a basal frequency of 100%, with increases and decreases in CBF reported as a percentage of this baseline frequency^6, 17^.

All experiments were conducted using air puff pressures of 55 mmHg. The air puff was always delivered to the middle of the transwell, 6 mm above the apical surface fluid, for 50 milliseconds. Once the cultures equilibrated to 26 **°**C, CBF during four 15-second intervals was recorded to determine a baseline frequency. The air puff was then delivered, and measurements were recorded every 15 seconds.

### ATP Release Assay

The apical surfaces of the ALI cultures were washed 3 times with PBS, and then incubated for 30 minutes with 50 μl of fresh PBS on the apical surface. Five minutes before the air puff, 10 μl of apical fluid was aspirated and diluted 10 times in PBS, and then boiled for 2 minutes to prevent degradation of ATP. At 15 seconds following the air puff, another 10 μl of apical fluid was aspirated, diluted 10 times in PBS, and then boiled for 2 minutes. Each ATP assay was carried out immediately following the experiment. The Enliten ATP assay kit (Promega, Madison WI, USA) was used for ATP quantification. A 10-μl blank, ATP standard, or sample solution was added to the wells of a 96-well microplate, and 100 μl of luciferin-luciferase (rL/L) reagent was added. A luminometer (Luminoskan Ascent 2.5; Thermo Labsystems, Franklin, MA, USA) quantified the ATP present in each sample using a 10-second integration time. As apical fluid volume varied depending on experimental conditions, all reported values are normalized to a 3.6 μl ASL volume, which has been experimentally determined to be the residual fluid on the apical surface following aspiration or clearance^63^. This correction is performed to approximate a physiologically relevant ATP concentration for use in future experimental paradigms; the volume of apical fluid used in our experiments for technical feasibility is higher than would be expected *in vivo*.

### qPCR

Wild-type, *Calhm1* knockout, and *Panx1* knockout cells grown for several weeks at an ALI were lysed using QiaShredder (Qiagen, cat# 79656) and total RNA was purified with an RNeasy Micro Kit (Qiagen, cat# 74004) according to standard protocols. Following purification, RNA was reverse-transcribed with an Invitrogen SuperScript III First-Strand cDNA Synthesis kit (Themo Fisher, cat# 18080051). Using this cDNA, real-time qPCR was carried out with SYBR® Green PCR Master Mix (Applied Biosystems, cat# 4309155) on an Applied Biosystems 7500 Real-Time PCR System (Applied Biosystems) for the analysis of β-actin, *Calhm1*, and *Panx1* genes. Primers were obtained from PrimePCR^™^ Assays and Controls (Bio-Rad) for *ActB* (β-actin) and *Panx1*, and *Calhm1* primers were obtained from Integrated DNA Technologies (F-primer: AGAACTTGCTCGCCTACTGG R-primer: CTCTTGAGAAAGGCGACCTG). β-actin was used as the house-keeping gene, and *Calhm1* and *Panx1* gene expression was determined by the 2^−ΔΔC^_T_ method^64^, which uses fluorescence and threshold values (C_T_) to compare the relative amount of target mRNA. PCR cycling conditions were 95 °C for 30 seconds, 65 °C for 30 seconds, and 72 °C for 45 seconds. All target expression levels were normalized relative to β-actin expression levels from paired cDNA samples.

### Statistics

GraphPad Prism5 (GraphPad Software, San Diego, CA, USA) was used for all statistical analyses, with results shown as means ± standard error. Kolmogorov-Smirnov (KS) testing for a Gaussian distribution was used to confirm normality of data and the use of parametric testing. An unpaired Student’s t-test was used for all comparisons unless otherwise noted, or if a non-parametric test was indicated by KS testing. A value of p < 0.05 was considered to be statistically significant.

### Data Availability

All data generated or analyzed during this study are available from the corresponding author on reasonable request.

## Electronic supplementary material

Supplementary Figures 1-3
