# Supplementary material for: CALHM1-Mediated ATP Release and Ciliary Beat Frequency Modulation in Nasal Epithelial Cells
Source: Sci Rep. 2017 Jul 27;7:6687. doi: 10.1038/s41598-017-07221-9 (PMC5532211; doi:10.1038/s41598-017-07221-9)
Supplement: Supplementary file 1 — Supplementary Figures 1-3 [file 41598_2017_7221_MOESM1_ESM.pdf]

CALHM1-Mediated ATP Release and Ciliary Beat Frequency Modulation in Nasal Epithelial Cells

Alan D. Workman, Ryan M. Carey, Bei Chen, Cecil J. Saunders Ph.D., Philippe Marambaud Ph.D., Claire H. Mitchell Ph.D., Michael G. Tordoff Ph.D., Robert J. Lee Ph.D., Noam A. Cohen M.D., Ph.D.

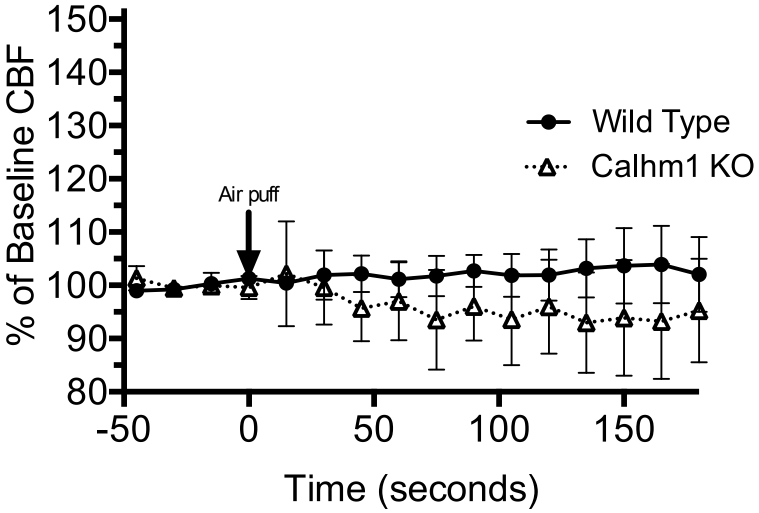

Supplemental Figure 1: CBF changes after a 55-mmHg air puff in nasal septal epithelial cell cultures from wild-type and *Calhm1* knockout cultures preincubated with 150 µM carbenoxolone. Symbols and bars are means ± SE of 3 cultures each.

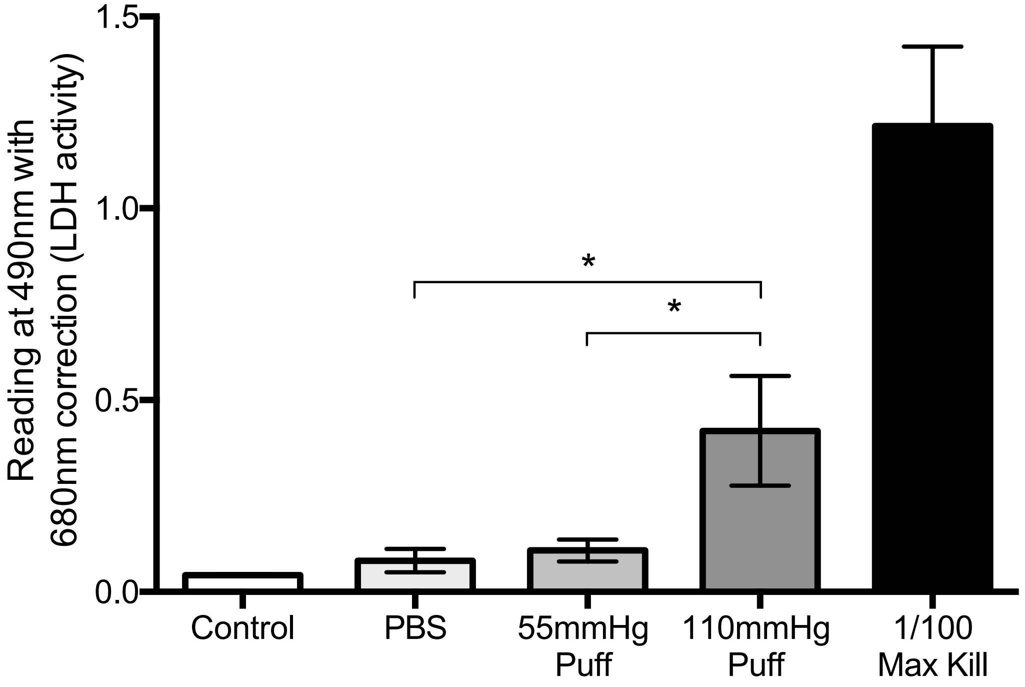

Supplemental Figure 2: LDH release following a 55mmHg or 110mmHg air puff in ALI cultures. Bars are means ± SE of 3-5 cultures each of LDH enzymatic assay reading. Maximum kill determined by LDH released following complete lysis of all ALI cells. *$p < 0.05$, ANOVA with Dunnett's multiple comparisons test.

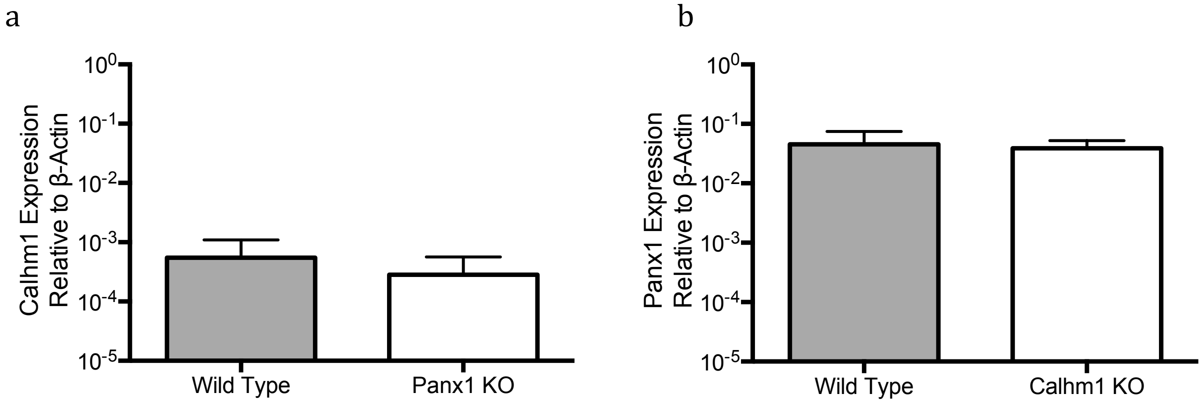

Supplemental Figure 3: (A) Relative expression of *Calhm1* transcripts in wild-type and *Panx1* knockout mouse cultures. (B) Relative expression of *Panx1* transcripts in wild-type and *Calhm1* knockout mouse cultures. All expression of target genes calculated relative to *ActB* (β-Actin) expression in paired samples. Bars are means + SE of 3-6 samples each.
